# Supplementary material for: Analysis and comparison of protein secondary structures in the rachis of avian flight feathers
Source: PeerJ. 2022 Feb 28;10:e12919. doi: 10.7717/peerj.12919 (PMC8893027; doi:10.7717/peerj.12919)
Supplement: Table S2 [file peerj-10-12919-s002.docx]

**Supplementary Table 2. Secondary structure calculation from flight feather rachis FT-IR spectra**

| **Assignments** | **Chicken** | | **Mallard** | | **Ibis** | |
| --- | --- | --- | --- | --- | --- | --- |
|  | **Cortex** | **Medulla** | **Cortex** | **Medulla** | **Cortex** | **Medulla** |
| β-turns (1,614 cm^-1^) | 8.86 ± 0.91% | 10.34 ± 0.10% | 6.71 ± 1.12% | 13.84 ± 1.88% | 6.24 ± 0.98% | 18.15 ± 1.10% |
| Parallel β-strand (1,627 cm^-1^) | 14.02 ± 1.28% | 19.45 ± 0.61% | 14.74 ± 0.95% | 20.23 ± 1.19% | 16.03 ± 1.48% | 22.99 ± 0.94% |
| Triple-helix (1,637 cm^-1^) | 12.49 ± 0.24% | 15.27 ± 1.13% | 13.41 ± 0.98% | 14.53 ± 1.37% | 11.78 ± 0.89% | 14.19 ± 0.78% |
| Unordered structure (1,648 cm^-1^) | 18.50 ± 0.25% | 17.81 ± 0.85% | 17.98 ± 0.89% | 15.50 ± 0.73% | 19.42 ± 2.21% | 14.43 ± 0.33% |
| α-helix (1,657 cm^-1^) | 8.40 ± 0.76% | 11.62 ± 0.68% | 8.63 ± 1.05% | 10.69 ± 0.03% | 9.91 ± 0.81% | 9.45 ± 0.85% |
| β-turns (1,668 cm^-1^) | 24.42 ± 0.33% | 14.32 ± 1.83% | 23.41 ± 0.44% | 15.48 ± 0.87% | 22.99 ± 1.26% | 13.06 ± 1.18% |
| β-sheets (Parallel β-strand, 1,683 cm^-1^) | 9.01 ± 0.21% | 9.04 ± 1.01% | 10.38 ± 0.74% | 7.04 ± 0.36% | 9.19 ± 0.44% | 5.97 ± 0.43% |
| β-sheets (Anti-parallel β-strand, 1,693 cm^-1^) | 4.30 ± 0.88% | 2.05 ± 0.28%% | 4.75 ± 0.89% | 2.68 ± 0.34% | 4.44 ± 0.77% | 1.77 ± 0.21% |

| **Assignments** | **Goshawk** | | **Owl** | | **Budgie** | | **Zebra finch** | |
| --- | --- | --- | --- | --- | --- | --- | --- | --- |
|  | **Cortex** | **Medulla** | **Cortex** | **Medulla** | **Cortex** | **Medulla** | **Cortex** | **Medulla** |
| β-turns (1,614 cm^-1^) | 8.82 ± 0.62% | 14.28 ± 1.14% | 8.09 ± 0.77% | 14.29 ± 1.14% | 9.68 ± 0.37% | 16.21 ± 0.89% | 6.83 ± 0.097% | 17.37 ± 0.21% |
| β-sheets (Parallel β-strand, 1,627 cm^-1^) | 17.29 ± 1.02% | 18.83 ± 0.11%% | 11.20 ± 0.19% | 18.83 ± 0.11% | 9.99 ± 1.23% | 20.36 ± 0.51% | 12.38 ± 3.01% | 20.81 ± 0.23% |
| Triple-helix (1,637 cm^-1^) | 11.22 ± 0.54% | 15.83 ± 0.55% | 14.52 ± 0.67% | 15.87 ± 0.55% | 15.57 ± 1.72% | 14.58 ± 0.43% | 15.65 ± 3.22% | 13.05 ± 0.32% |
| Unordered structure (1,648 cm^-1^) | 20.54 ± 1.13% | 15.58 ± 0.52% | 19.53 ± 0.93% | 15.58 ± 0.52% | 19.69 ± 1.29% | 15.60 ± 0.59% | 13.57 ± 2.15%% | 15.13 ± 0.46% |
| α-helix (1,657 cm^-1^) | 11.66 ± 2.66% | 8.57 ± 0.55% | 12.07 ± 0.15% | 8.57 ± 0.55% | 10.09 ± 0.76% | 8.60 ± 1.14% | 9.26 ± 0.33% | 8.22 ± 0.42% |
| β-turns (1,668 cm^-1^) | 18.08 ± 0.79% | 17.59 ± 0.37% | 18.28 ± 0.24% | 17.59 ± 0.37% | 21.23 ± 0.62% | 14.58 ± 1.84% | 26.22 ± 0.12%% | 14.29 ± 0.94% |
| β-sheets (Parallel β-strand, 1683 cm^-1^) | 9.68 ± 1.20% | 7.57 ± 0.50% | 12.37 ± 0.17% | 7.57 ± 0.50% | 8.89 ± 1.29% | 7.22 ± 0.53% | 10.26 ± 0.17% | 8.20 ± 0.52% |
| β-sheets (Anti-parallel β-strand, 1,693 cm^-1^) | 2.27 ± 0.69% | 1.70 ± 0.26% | 3.94 ± 0.31% | 1.70 ± 0.26% | 4.87 ± 0.47% | 2.57 ± 0.20% | 5.84 ± 0.70% | 2.92 ± 0.29% |

The ratio between the secondary structures peaks area and the area of the entire Amide I band, as a percentage, are shown in the table. N = 3 spectra for each sample.
