# Supplementary figures and images for: Analysis and comparison of protein secondary structures in the rachis of avian flight feathers
Source: PeerJ. 2022 Feb 28;10:e12919. doi: 10.7717/peerj.12919 (PMC8893027; doi:10.7717/peerj.12919)

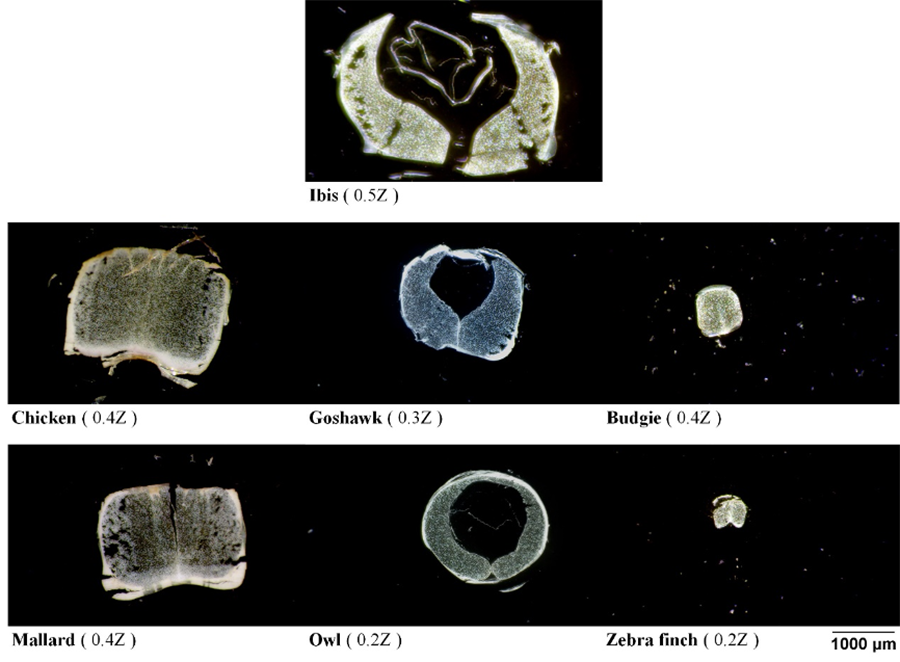

Supplement: Figure S1 [file peerj-10-12919-s003.png]

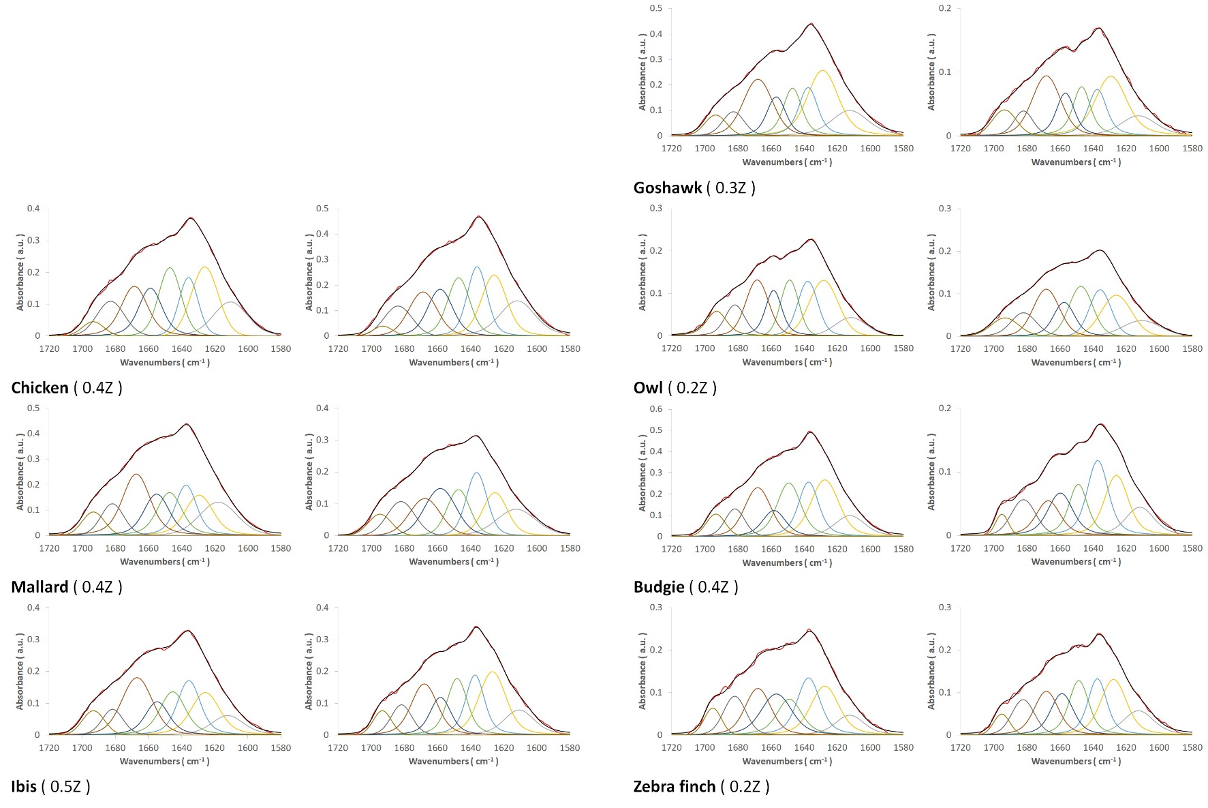

Supplement: Figure S2 — The black line is the fitted spectrum, and the red line is the normalized original spectrum. [file peerj-10-12919-s004.png]

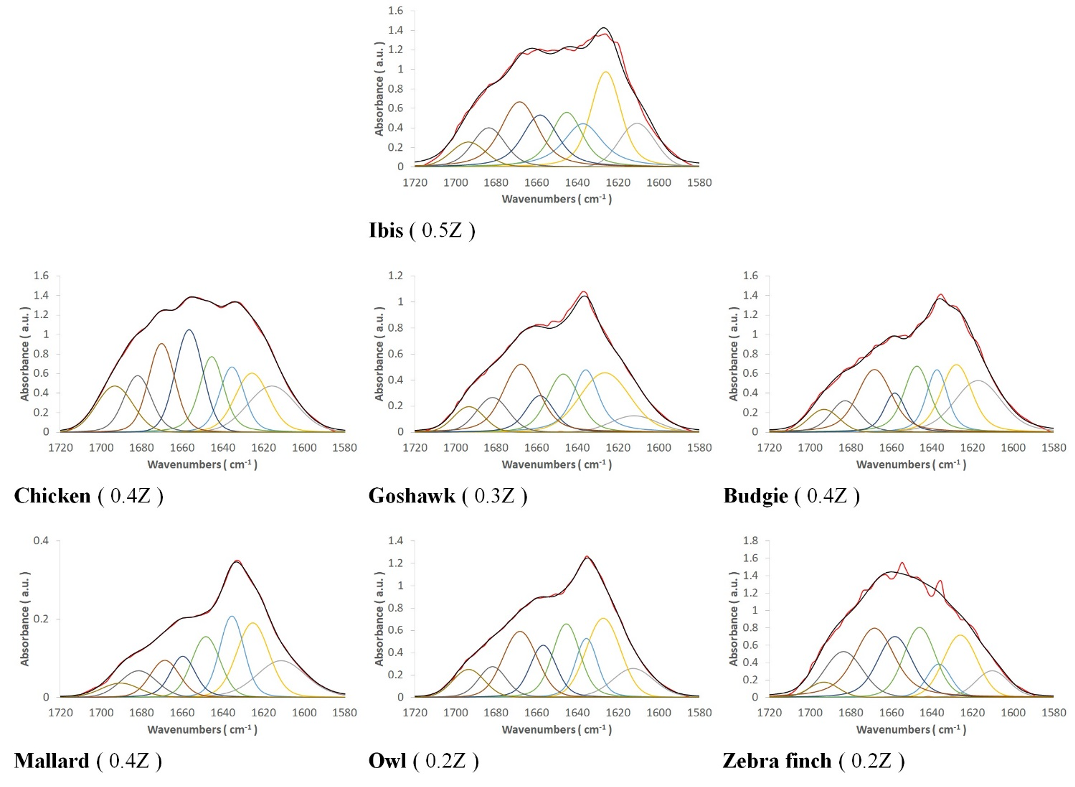

Supplement: Figure S3 — The black line is the fitted spectrum, and the red line is the normalized original spectrum. [file peerj-10-12919-s005.png]

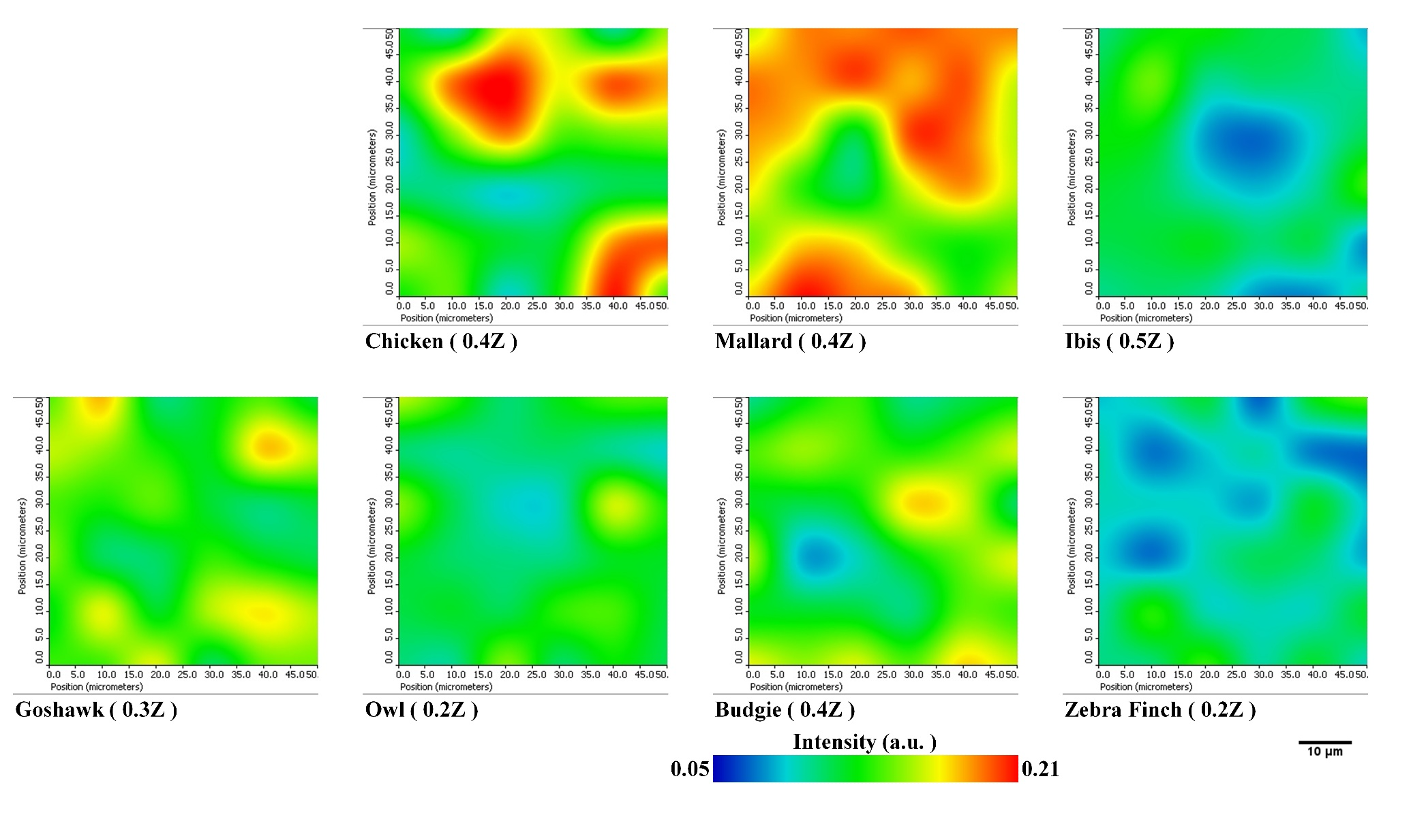

Supplement: Figure S4 [file peerj-10-12919-s006.png]

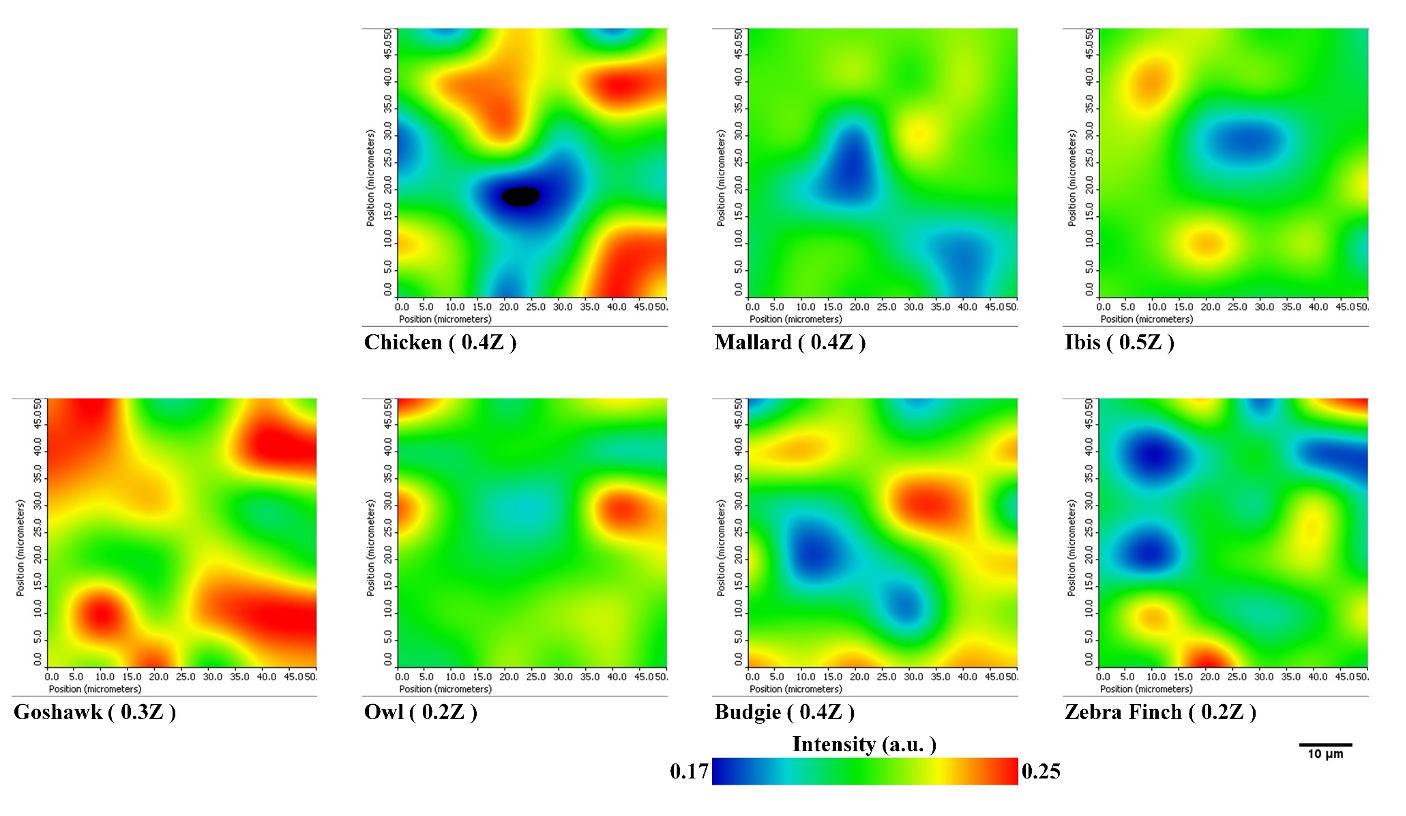

Supplement: Figure S5 [file peerj-10-12919-s007.png]

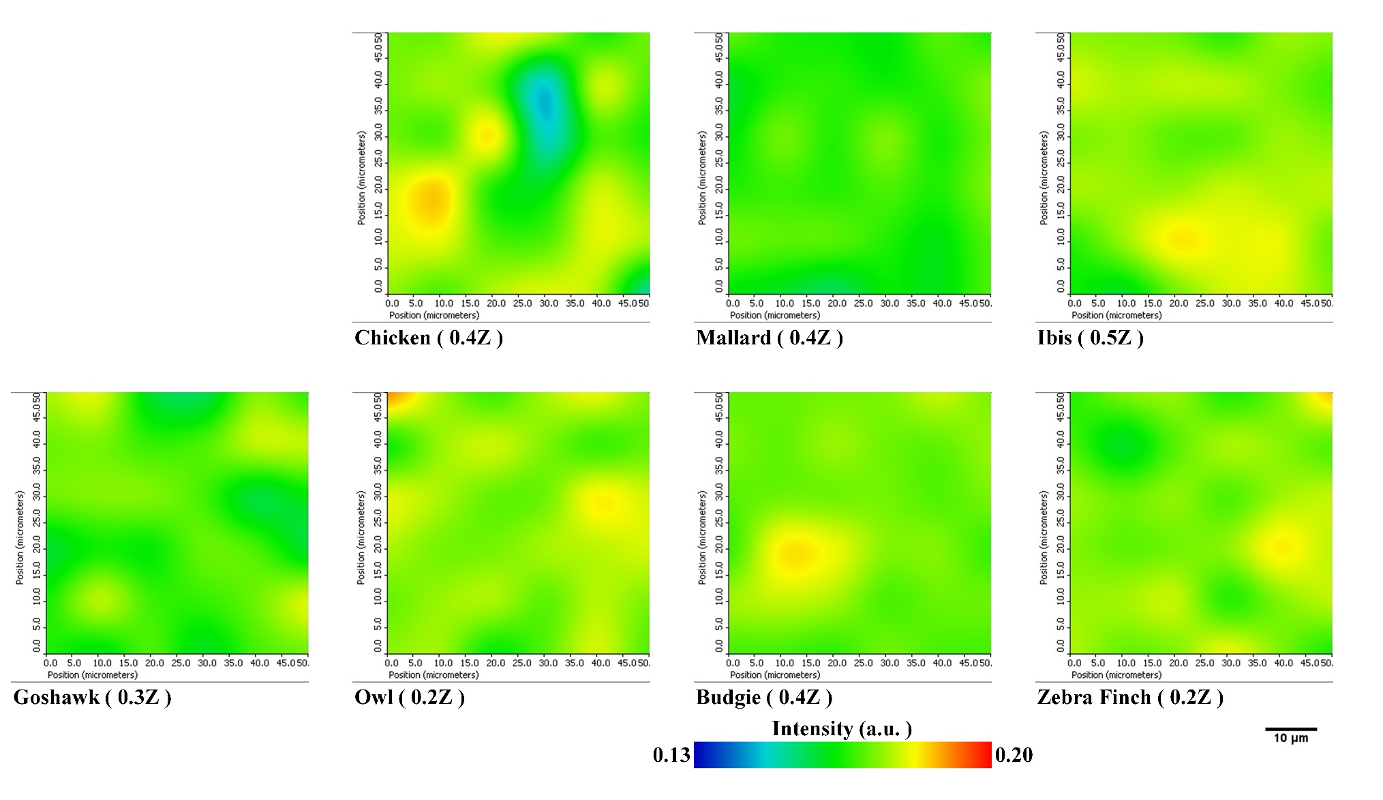

Supplement: Figure S6 [file peerj-10-12919-s008.png]

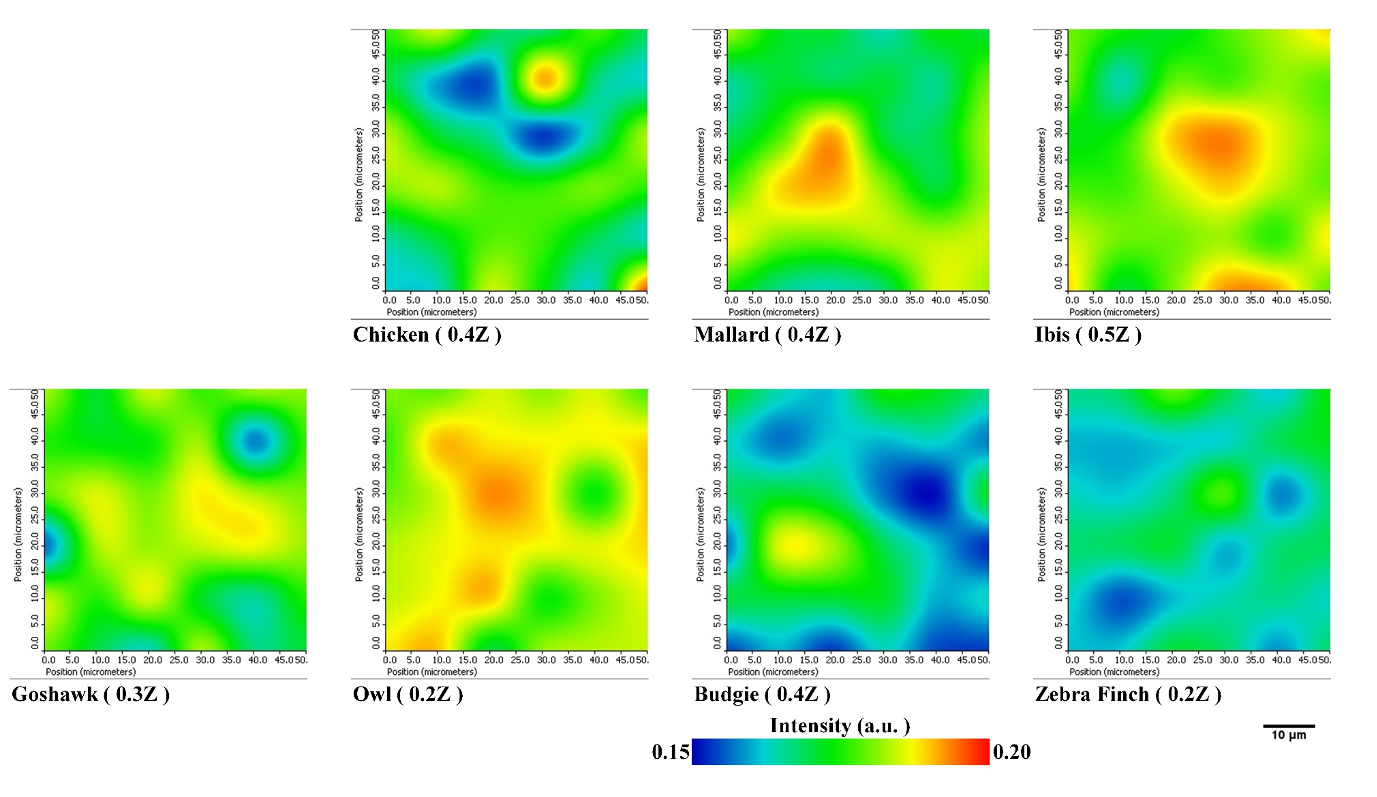

Supplement: Figure S7 [file peerj-10-12919-s009.png]

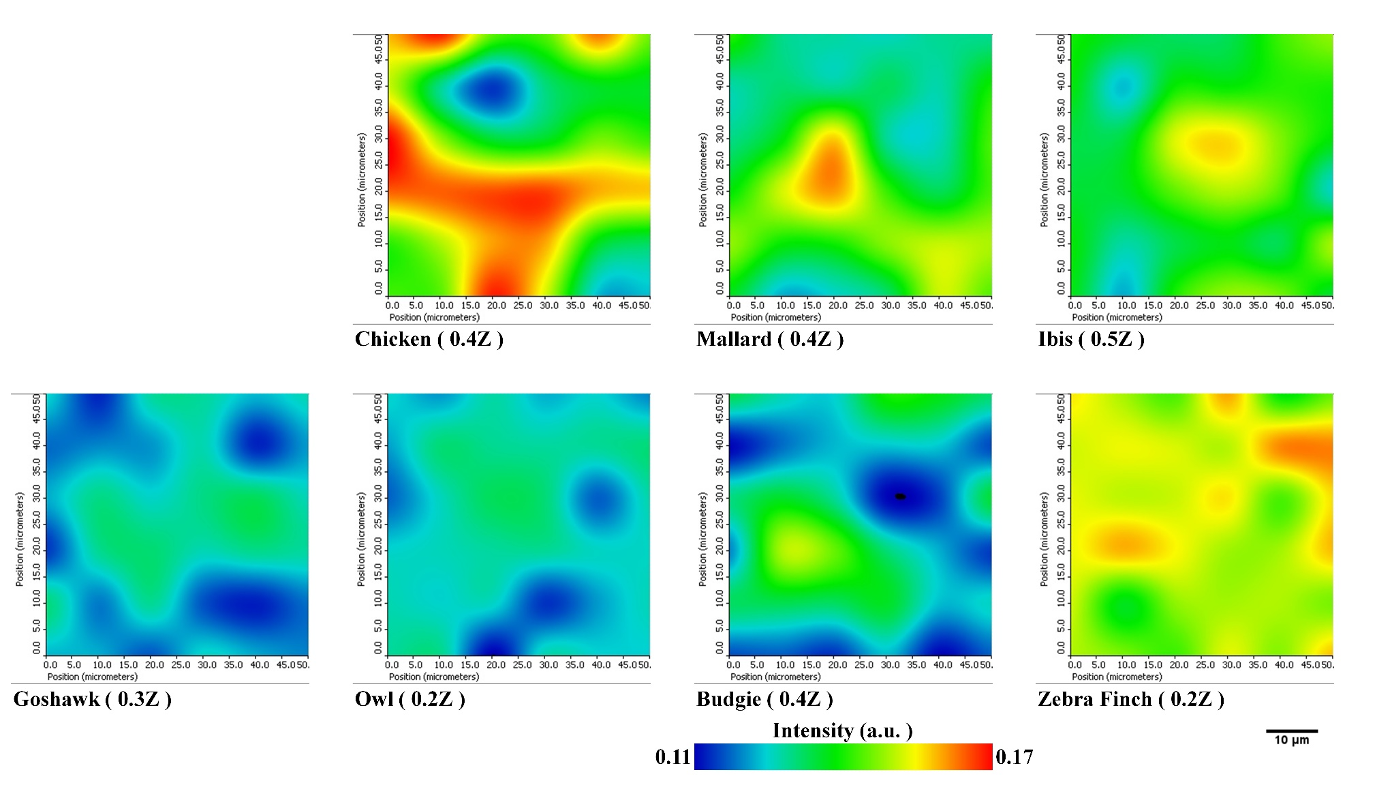

Supplement: Figure S8 [file peerj-10-12919-s010.png]

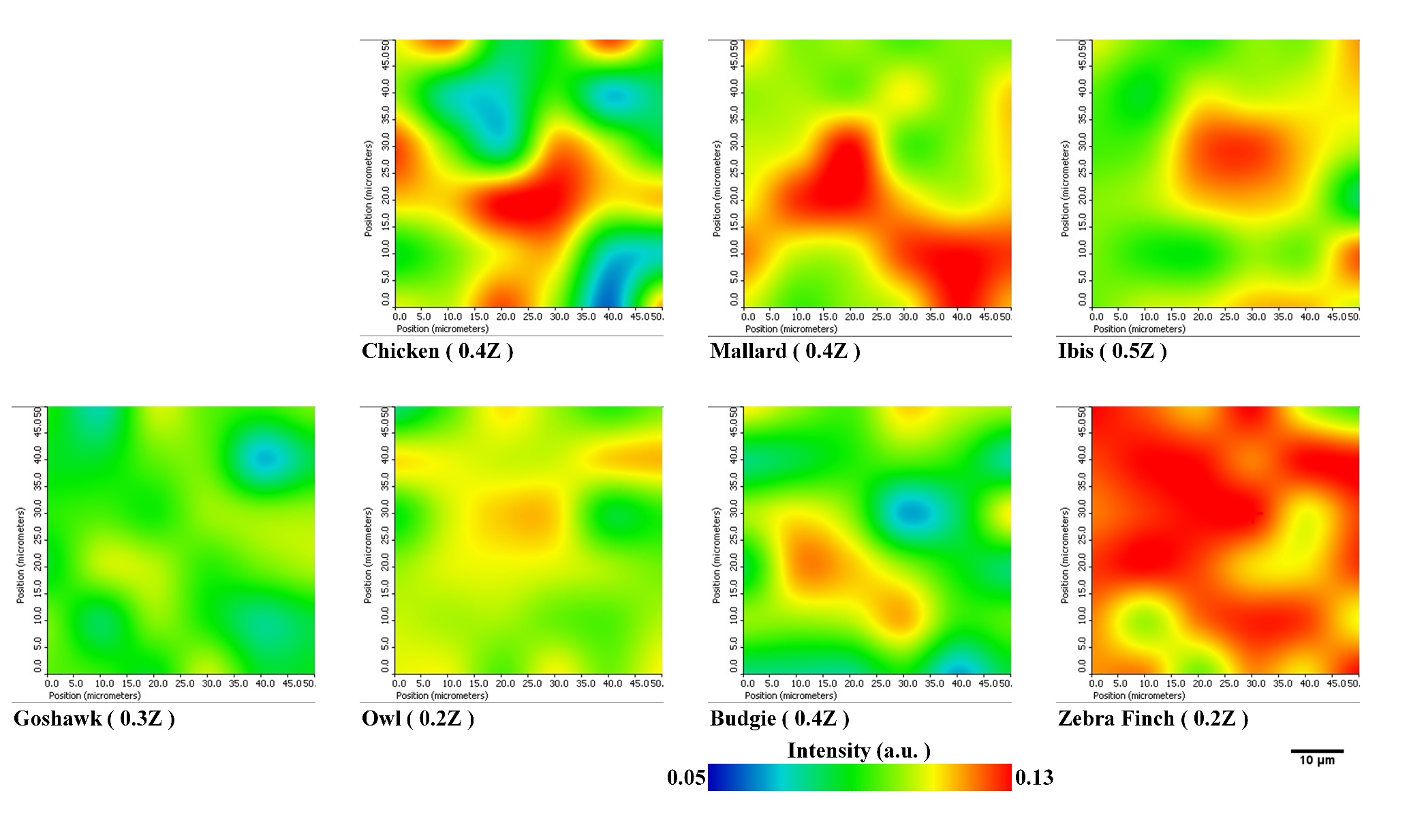

Supplement: Figure S9 [file peerj-10-12919-s011.png]

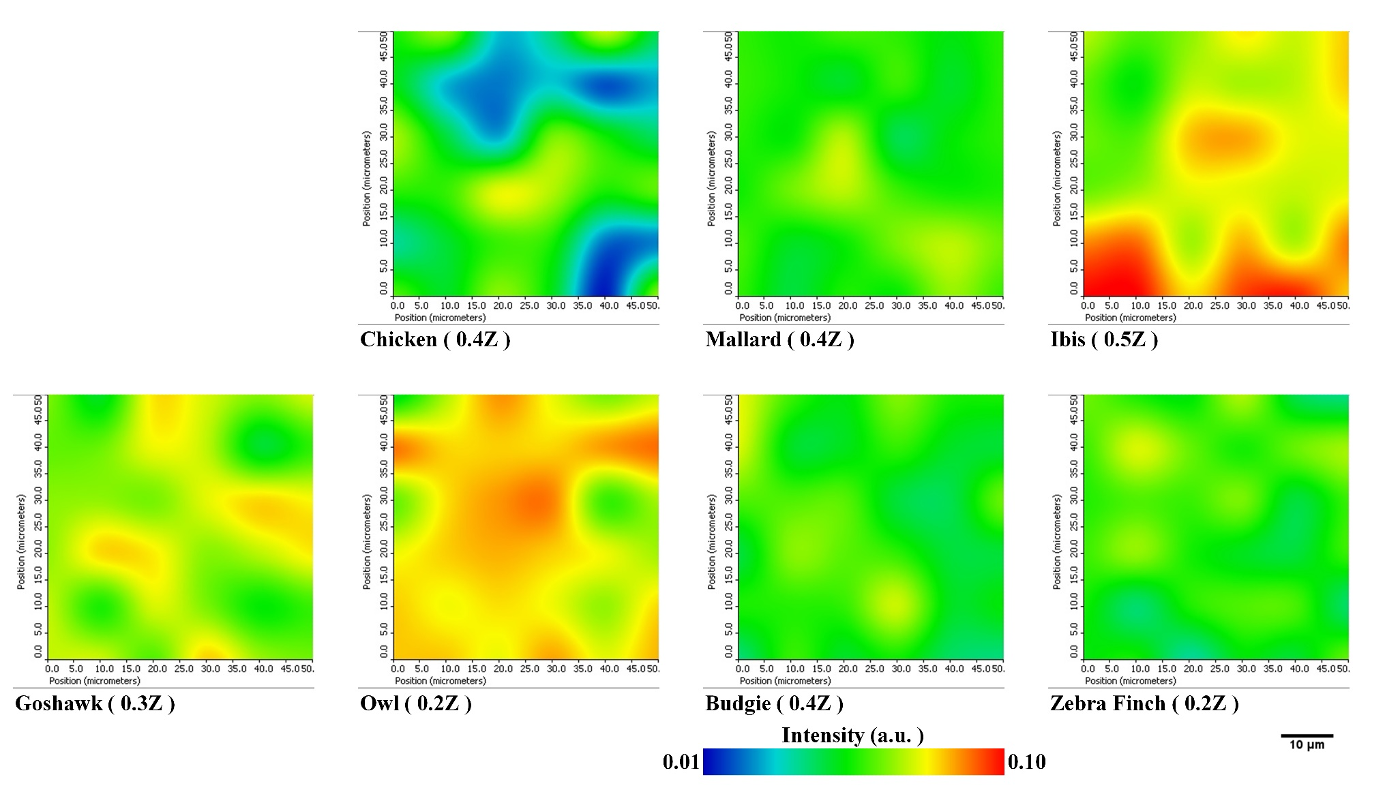

Supplement: Figure S10 [file peerj-10-12919-s012.png]
